# Impact of Rater Change on MMSE Data. A Post‐Hoc Analysis

**DOI:** 10.1002/alz.088899

**Published:** 2025-01-09

**Authors:** Alan Kott, Xingmei Wang, David S. Miller

**Affiliations:** ^1^ Signant Health, Prague Czech Republic; ^2^ Signant Health, Blue Bell, PA USA

## Abstract

**Background:**

Rater change is inevitable in often lengthy clinical trials in Alzheimer’s disease. Other groups have previously assessed the impact of rater change on data variability. Their conclusions varied, possibly due to differing methodologies (e.g. ‐ comparing actual vs. absolute change; analyzing data per trial and visit or by combining all trials and visits). Here, we analyze the impact of rater change of MMSE data ‐ using both actual and absolute change ‐ looking only between Screening and Baseline visits.

**Method:**

MMSE and MMSE change from Screening data were obtained from 11.084 Baseline assessments collected across 13 Alzheimer’s disease clinical trials. Data were broken into 2 groups by the presence of rater change between Screening and Baseline. Two regression models were fitted to the data. In the first model the dependent variable was the MMSE actual change from Screening to Baseline, in the second model the MMSE absolute change was tested. Rater change, clinical trial and their interaction were used as predictors and MMSE Baseline value entering in a cubic form as a covariate.

**Result:**

In the first model using actual MMSE change, neither rater change, nor the interaction between protocol and rater change was significant. Removing the interaction term, rater change decreased the MMSE change by 0.1 MMSE points (p = 0.06). In the second model, the effect of rater change differed from study to study. Among studies that showed significant rater change effect, the absolute MMSE change increased by 0.46 points on average.

**Conclusion:**

Our results indicate that rater change may indeed have an impact on MMSE data but the impact is inconsistent. While the actual change was affected only insignificantly, the absolute change as a measure of variability was significantly increased. Substantial differences were observed between individual studies and in only 6 of the 13 studies, rater change significantly increased MMSE absolute change. Given the results, rater change should be considered a risk factor to assessment reliability. Therefore, reasonable efforts should be taken to minimize both its occurrence and its impact on study data.

